# The Role of Seagrass Traits in Mediating *Zostera noltei* Vulnerability to Mesograzers

**DOI:** 10.1371/journal.pone.0156848

**Published:** 2016-06-03

**Authors:** Begoña Martínez-Crego, Pedro Arteaga, Fiona Tomas, Rui Santos

**Affiliations:** 1 Centre of Marine Sciences (CCMAR), Faro, Portugal; 2 Mediterranean Institute for Advanced Studies (IMEDEA-CSIC), Esporles, Balearic Islands, Spain; 3 Department of Fisheries and Wildlife, Oregon State University, Corvallis, Oregon, United States of America; College of Charleston, UNITED STATES

## Abstract

Understanding how intra-specific differences in plant traits mediate vulnerability to herbivores of relevant habitat-forming plants is vital to attain a better knowledge on the drivers of the structure and functioning of ecosystems. Such studies, however, are rare in seagrass-mesograzer systems despite the increasingly recognized relevance of mesograzers as seagrass consumers. We investigated the role and potential trade-offs of multiple leaf traits in mediating the vulnerability of the seagrass *Zostera noltei* to different mesograzer species, the amphipod *Gammarus insensibilis* and the isopod *Idotea chelipes*. We worked with plants from two different meadows for which contrasting chemical and structural traits were expected based on previous information. We found that plants with high vulnerability to mesograzers (i.e. those preferred and subjected to higher rates of leaf area loss) had not only higher nitrogen content and lower C:N, fibre, and phenolics, but also tender and thinner leaves. No trade-offs between chemical and structural traits of the seagrass were detected, as they were positively correlated. When leaf physical structure was removed using agar-reconstituted food, amphipod preference towards high-susceptibility plants disappeared; thus indicating that structural rather than chemical traits mediated the feeding preference. Removal of plant structure reduced the size of isopod preference to less than half, indicating a stronger contribution of structural traits (> 50%) but combined with chemical/nutritional traits in mediating the preference. We then hypothesized that the high environmental nutrient levels recorded in the meadow exhibiting high susceptibility modulate the differences observed between meadows in seagrass traits. To test this hypothesis, we exposed low-vulnerability shoots to eutrophic nutrient levels in a 6-week enrichment experiment. Nutrient enrichment increased *Z*. *noltei* nitrogen content and lowered C:N, fibre, and phenolics, but had no effect on structural traits. Overall, our findings help to better understand the trait-mediated seagrass susceptibility to mesograzers and reinforce the increasingly recognized role of structural defences against herbivory.

## Introduction

Herbivory is a key factor shaping community structure and functioning through the control of plant abundance [1, 2], with this control being particularly intense in marine environments [3]. Vulnerability to herbivores is inversely linked to the several resistance strategies that plants and seaweeds have evolved to lessen the impact of herbivory by affecting herbivore feeding behaviour and fitness [4, 5]. Such strategies encompass diverse nutritional, chemical, and structural traits that play a vital role in mediating plant palatability to herbivores. Despite the critical role of herbivory in ecological communities, there are many gaps in our understanding of the traits that are most strongly associated with plant resistance against herbivores, and the relative importance of different types of traits involved in anti-herbivory defence [6]. Moreover, while interspecific differences in vulnerability to herbivory have been widely studied in terrestrial, freshwater, and marine systems (e.g. [7, 8, 9]), comparatively less information is available regarding differences in vulnerability within single species of marine producers to different herbivore species (but see e.g. [10, 11]).

Nutritional content or levels of chemical defences have been commonly pointed out as the most important determinants of food choice by most herbivores in terrestrial, freshwater and marine systems [12, 13, 14, 15]. Nevertheless, the role of chemical traits is not straightforward. For instance, the deterrent effect of secondary metabolites such as phenolics is highly dependent on the identity of both, the herbivore and the specific group of phenolic compounds considered [16, 17]. More recently, the relevant role of structural defences (e.g. [11, 18, 19, 20]) and of intra-specific variation in plant morphology ([21, 22, 23]) in determining the herbivore feeding preferences for both vascular plants and algae has been highlighted in several studies. Structural defences such as leaf toughness and thickness, or fibre accumulated in plant cell walls, reduce not only leaf mechanical fracture, but also ingestion or digestibility [24, 25].

Although chemical and structural traits are not mutually exclusive defences of plants against herbivory, resource limitation and competition between defensive functions have been suggested to drive a physiological trade-off between these two kinds of defences [26, 27]. The basis of this trade-off relies on the assumption that defences divert the limited pool of available resources from growth according to plant-defence theories unified within the growth-differentiation balance hypothesis [28, 29]. More recent hypotheses, however, consider defences as a suite of co-varying traits acting in concert (i.e. defence syndromes) rather than as a single attribute ruled by trade-offs [30, 31, 32]. Our understanding about how these multiple traits interact and influence resistance against herbivores is still scarce.

Plastic chemical and structural traits, and subsequently any potential trade-off between them, may vary through plant ontogeny [33], between reproductive and vegetative tissues [11, 34], between species or populations [35, 36], and in space or time across resource gradients [28, 37, 38, 39, 40]. Such variation often has a strong influence on herbivore preference for particular plant parts, individuals, or populations (e.g. [11, 19, 37, 38, 41]). Particularly, the growth-differentiation balance hypothesis predicts that plants growing in low-nutrient environments should be better defended due to re-growth constraints [29]; thus suggesting that nutrient availability can be particularly relevant in shaping intra-specific differences in plant traits and vulnerability to herbivory across sites or populations.

Seagrasses are marine vascular plants that form highly productive meadows worldwide. Seagrass beds provide critical ecosystem services to the overall function of coastal marine ecosystems [42], but they are drastically declining across the globe due to human impacts such as coastal eutrophication [43]. Exceptionally high herbivory rates of large grazers such as sea urchins, fishes, or turtles have been identified as a major biological driver of seagrass decline in altered systems, often linked to nutrient enrichments or predator overfishing [42, 44, 45, 46]. Historical overfishing of large grazers has led to the dominance of mesograzers (i.e. small invertebrate grazers; mostly gastropods, amphipods and isopods) and some fish species in the herbivory pathway in many areas worldwide [47]. Furthermore, increasing evidence suggests that mesograzers can directly consume or cause the loss of substantial quantities of seagrass production [48, 49, 50, 51, 52]. However, we know little regarding whether different mesograzer species respond equally or differently to changes in multiple seagrass traits.

Mesograzer sensitivity to chemical defences is ambiguous, as certain amphipods and isopods tolerate the brown algal phlorotannins, an extensively studied kind of phenolic compounds that deter the feeding of fishes and sea urchins (reviewed by [16]). This tolerance has led to the classic hypothesis in which Hay et al. [53, 54] proposed that mesograzers selectively choose chemically defended seaweeds as habitat and food in order to lower predation risk to omnivorous fishes. More recently, strong interspecific differences have been reported between mesograzer species in their ability to induce seagrass chemical defences that deter further consumption [55]. At the same time, fine scale structural aspects of food such as toughness and thickness are expected to have a strong influence on small animals that need to scrape or bite through the full thickness of the leaf [56]. This may render mesograzers especially sensitive to structural defences. However, the role of nutritional, chemical, and structural traits in mediating seagrass vulnerability to mesograzers has only been scarcely investigated, particularly in *Zostera marina* or *Cymodocea nodosa* and with a single mesograzer species (e.g. [36, 57]). This understanding is particularly relevant nowadays, when anthropogenic nutrient enrichments of coastal waters may shift seagrass biochemical and structural traits by altering allocation of resources to growth, storage, and defences.

In this study, we examine the role of seagrass traits in modulating vulnerability to mesograzer herbivory using a suite of feeding assays. Particularly, we address the following questions: (1) Which seagrass traits (chemical or structural) are best related to *Zostera noltei* vulnerability to different mesograzer species? (2) Is there any trade-off between multiple traits in providing *Z*. *noltei* resistance against mesograzers? (3) May coastal eutrophication (i.e. nutrient enrichment) modify seagrass traits mediating *Z*. *noltei* vulnerability to mesograzers?

## Materials and Methods

### Study site and organisms

We collected low-intertidal *Zostera noltei* plants and their associated mesograzers within the Ria Formosa lagoon (37°00´N, 7°53´W, NE Atlantic, Southern Portugal). The seagrass *Z*. *noltei* is an important fast-growing facilitator species that forms extensive beds in protected bays, coastal lagoons and estuaries along the Northeast Atlantic and Mediterranean, Black, Caspian, and Aral Seas [58]. To investigate the underlying traits that determine plant-specific vulnerability to mesograzers, we worked with plants from two populations with contrasting seagrass traits and with two mesograzer species, the amphipod *Gammarus insensibilis* (mean ± SE: 1.5 ± 0.06 cm length, n = 32) and the isopod *Idotea chelipes* (1.3 ± 0.04 cm length, n = 30). Both mesograzer species are widely distributed and use seagrass meadows for refuge and food (e.g. [59, 60]). Permission for sampling at the Ria Formosa was provided by the Portuguese ICNF (Instituto da Conservação da Natureza e das Florestas). No protected species were sampled.

Prior to the experiments, environmental and biotic conditions of seagrass meadows across Ria Formosa were monitored during low (spring and neap) tides in spring-summer seasons of 2011 and 2013 and two meadows for which we expected contrasting seagrass traits were selected (hereafter PRAIA and QUINTA). QUINTA meadow had ca. 4-fold higher nutrient levels in the water column than PRAIA, including nitrate (mean ± SE: 1.0 ± 0.2 and < 0.01 μM, respectively; Mann-Whitney U = 3.5, p = 0.004, n = 15), ammonium (3.4 ± 0.4 and 0.7 ± 0.2 μM, respectively; U = 11, p < 0.001, n = 15), and phosphate (1.3 ± 0.1 and 0.5 ± 0.1 μM; t = -6.7, p < 0.001, n = 15). QUINTA also showed significantly higher algal (*Ulva* spp.) accumulation (38 ± 12% of meadow surface covered, n = 9) than PRAIA (*Ulva* spp. absent; Mann-Whitney U = 9.0, p = 0.002). Other biotic and environmental factors monitored did not significantly differ between meadows. Both, QUINTA and PRAIA meadows, showed similar epiphyte load on seagrass leaves (0.8 ± 0.2 and 0.4 ± 0.1 mg cm^-2^, respectively; t = -1.7, p = 0.14, n = 4) and levels of fish herbivory (13 ± 5 and 14 ± 5% of leaves with bite marks shoot^-1^; Mann-Whitney U = 199, p = 0.99, n = 20), as well as similar levels of salinity (39.4 ± 0.2 and 38.9 ± 0.2 psu), light (5304 ± 249 and 5691 ± 199 lum ft^-2^), temperature (28.2 ± 0.2 and 28.8 ± 0.2°C), and hydrodynamics (as reflected by the sediment size: ϕ = 5.7 ± 0.5 and 5.6 ± 0.2; t = -0.15, p = 0.88, n = 5). Seawater nutrient concentrations were analysed using a loop-flow analyser (μMac-1000; Systea, Anagni, Italy), light and temperature were measured using Onset HOBO loggers, and salinity using a refractometer. *Ulva* spp. cover was measured within quadrats of 0.5 m x 0.5 m placed every 5 m along 15 m transects. Canopy height was measured within each quadrat ignoring the 20% tallest leaves. Fish herbivory was quantified in 3–4 shoots within each quadrat.

### Seagrass vulnerability: Mesograzer feeding assays

To experimentally examine between-meadow differences in seagrass vulnerability to different mesograzer species, we conducted a suite of two-choice and no-choice feeding assays in April 2013. Assays were run in an outdoor seawater flow-through system at the Ramalhete field station (CCMAR), where individual grazers were placed in 500 ml plastic cups with two parallel windows covered by a 1.5 mm mesh to allow water flow. Mesograzers were acclimated for 24–48 hours prior to starting each assay, during which time they were fed the palatable alga *Ulva* spp. to avoid any interference of previous food or starving on their foraging behaviour.

*Z*. *noltei* vulnerability in terms of feeding choices between PRAIA and QUINTA plants was investigated using paired preference assays. Amphipods and isopods were offered in 20 initial replicates a choice between comparable pieces of individual leaves (ca. 2.5 x 0.2 cm^2^), which were of similar age and cleaned of epiphytes. Each replicate consisted of one suction pad with to parallel and labelled incisions in which each choice was inserted leaving ca. 2.3 cm of both tissue types above the insertion. Replicates where grazers failed to feed (no area change and no bite marks visible) were discarded for statistical analyses as uninformative, a standard procedure in feeding preference experiments (e.g. [11, 41, 61, 62]). Assays lasted 2 days or until ca. 50% of any choice was consumed, whichever came first. Leaf area was measured at the beginning and at the end of each assay through image analysis using the program Adobe Photoshop CS3. Mesograzer preference was quantified by comparing leaf area consumed of each choice in a feeding assay. In addition to the consumed biomass, mesograzers increased the loss of seagrass tissues by facilitating the breakage of leaf fragments that were not consumed, as has been reported for algae thallus [63]. To take this loss into account, data of both area consumed and area lost to mesograzers were measured. Lost area referred to pieces of plant that were broken off from the leaf due to mesograzer bites but remained uneaten at the end of the feeding assay. Detached leaf pieces were identified just before breaking off from the leaf and afterwards allocated in the corresponding insertion of the suction pad. During monitoring of feeding assays, we observed that when mesograzers grazed the leaf it was evidenced in form of area loss, and so we are confident that measurements on area adequately represent feeding patterns. Ten control replicates (i.e. paired seagrass pieces of each type in cups with no grazer) were used to account for potential changes in leaf area due to factors other than herbivory. No change in their area was detected and they were thus not considered in the statistical analyses.

When a preference with fresh leaves was observed, agar-reconstituted food was used to examine the role of morphological/structural traits and chemical/nutritional defences in determining the feeding choices between PRAIA and QUINTA plants following a method adapted from Hay et al. [64]. Freeze-dried seagrass leaves were ground to a homogenous fine powder to remove plant structural traits while keeping chemical traits intact, and then reconstituted with an agar solution (combining 0.2 g of seagrass with 0.4 g of agar in 8 mL of distilled water). The seagrass-agar mixture was poured over a mosquito mesh, flattened between two glass panels to obtain a uniform thickness, and allowed to cool for 1 h in a refrigerator. The solidified mixture adhered to the mesh was cut into agar rectangles of identical size and shape (1.0 x 1.7 cm^2^), which were offered to a single mesograzer in 20 initial replicates. Assays lasted 3 days or until ca. 50% of any choice was consumed, whichever came first. Set-up and all other conditions were identical to those in the assays using fresh seagrass pieces, but reconstituted food was offered for a longer period than fresh food because more time was needed for area changes to occur in this type of food. Consumption was measured by counting the number of mesh squares that were cleared of food. Ten control replicates (i.e. paired agar strips in cups with no grazer) did not change in the number of squares covered and were thus not considered further in the analysis.

We also quantified rates of leaf area consumption and loss to mesograzers of PRAIA and QUINTA plants in no-choice assays. Each type of seagrass was offered *ad libitum* for 2 days in 10 and 12 replicates for isopods and amphipods, respectively, with the same setup as that described above. Consumption and loss rates were expressed in cm^2^ day^-1^ and were used to estimate grazing impact as indicator of seagrass vulnerability. Consumption rates were also used to examine the existence of any compensatory feeding driven by differences in the nutritional quality of food.

Differences in feeding preferences between choices for all paired assays were analysed using paired *t* tests. When a significant preference was detected in assays using fresh food and the same trend was observed with reconstituted food, the effect sizes for each type of assay were compared in order to better understand the relative contribution of chemical/nutritional and structural traits (i.e. null hypothesis of 50:50% contribution of chemical and structural traits when effect size with agar-reconstituted food is half the effect size with fresh food). Effect sizes were separately calculated for each type of assay as the difference between consumption of PRAIA and QUINTA material and compared using a non-parametric Mann-Whitney test because data were not normally distributed and had unequal variances. Unpaired *t* tests were used to compare differences in rates of consumption and loss (i.e. no-choice assays), since they came from independent assays. We checked all data for normality and homogeneity of variances, and when necessary data were log-transformed to meet normality.

### Seagrass leaf traits

To examine which seagrass traits relate more strongly to seagrass vulnerability to mesograzers and how they correlate to each other, we simultaneously collected plants for feeding assays and for chemical and structural analyses. Chemical traits were measured in four replicates of pooled leaf material (5 randomly collected shoots) frozen *in situ* using liquid nitrogen after removing epiphytes, and then freeze-dried and ground to fine powder. Carbon and nitrogen content in leaves were analysed using an elemental analyser (Carlo-Erba, Milan, Italy). Total phenolics were extracted in methanol 50% for 24 h under constant agitation at 4°C and determined with a spectrophotometer following a modified Folin-Ciocalteu assay using chlorogenic acid as standard (modified from Bolser et al. [62]). Insoluble fibre was determined as the remaining dry weight after boiling the sample in neutral detergent for 1 h and successively washing with distilled water, ethanol, and acetone following a method from de los Santos et al. [65].

As structural traits, we measured leaf thickness, cross-sectional area, and breaking force at 2–5 cm above the sheath junction in 12–14 fully developed and healthy leaves of similar age from independent shoots. Because amphipods and isopods bite the leaf surface until breaking through the complete leaf thickness, the vertical force needed to penetrate leaf tissue was measured using a penetrometer following a method commonly used in ecological studies (e.g. [66, 67]). Structural traits were measured in both, fresh and frozen/thawed leaves, which allowed confirming the suitability of the latter for comparative purposes in the nutrient enrichment experiment (for which only frozen/thawed leaves were available; see also [11]). Prior to the breaking test, leaf width and thickness were measured with a digital calliper (Mitutoyo, precision ± 0.01 mm) and the cross-section area was calculated. Leaf thickness was also considered as a separate component of leaf resistance because it can be relevant for mesograzers that have to bite through the full thickness of the leaf [31, 56].

For each individual seagrass trait, we analysed differences between PRAIA and QUINTA plants using unpaired *t* tests. When necessary, data were log-transformed to meet normality. To assess relationships between *Z*. *noltei* leaf traits, we ran principal component analysis (PCA) using both PRAIA and QUINTA plants as replicates after randomly pairing and averaging replicates of structural traits to equal replicates of chemical traits (n = 8). PCA was run from the correlation matrix, in which the variable scores reflect the correlation to the components and the angles between variables are proportional to their covariances. Significance of PCA results was further confirmed by Pearson or Spearman correlations between each pair of variables.

### Nutrient-enrichment effects on seagrass traits

To assess if or how nutrient-induced changes affect seagrass traits, we conducted a 6-week nutrient-enrichment experiment with PRAIA plants at the Ramalhete field station during August. This time span is enough for *Z*. *noltei* to grow whole new leaves and experience nutrient-driven changes in seagrass traits [68]. *Z*. *noltei* plants were collected in natural densities with their natural sediments and they were allocated in mesocosms (tanks of 110 L) in an outdoor open system (i.e. around 500 shoots within each replicate mesocosm). Sand-filtered seawater from the lagoon was independently supplied to each mesocosm at a rate of 240 L h^–1^. Plants were left to acclimate for 4 days before 4 replicate mesocosms were randomly assigned to unfertilized and nutrient-enriched treatments. In the enriched mesocosms, a solubilised mixture of ammonium nitrate and monoammonium phosphate fertilizers was added directly into the water column using a multi-channel dosing pump. Nutrient levels in the control treatment encompassed the natural values found in the lagoon (mean ± SE: 0.3 ± 0.1 μM ammonium, 2.3 ± 0.2 μM nitrate, and 0.3 ± 0.02 μM phosphate; n = 24), while the enriched treatment exhibited eutrophic nutrient levels (74 ± 1.4 μM ammonium, 44 ± 1.7 μM nitrate, and 3.9 ± 0.1 μM phosphate; n = 29) that are within the lower range of levels found in the lagoon in *Z*. *noltei* meadows close to urban wastewater discharges [69]. Eutrophic conditions, with nutrient levels higher than those measured in our study sites, were chosen for enriched mesocosms in order to increase the probability of significant responses on seagrass traits. To monitor nutrient levels, water samples were analysed weekly using a loop-flow analyser (μMac-1000; Systea, Anagni, Italy).

At the end of the enrichment experiment, we quantified several nutritional, chemical and structural traits in plants collected from each mesocosm using the same procedures as described previously. Leaf structural traits were measured on 6–12 fully developed leaves from independent shoots within each mesocosm and the mean value was used as an independent replicate. We checked all data for parametric assumptions of normality and homogeneity of variances. For each individual seagrass trait, we analysed differences between unfertilized and nutrient-enriched plants using unpaired *t* tests or using Welch´s *t* tests when data had unequal variances. Links among traits were investigated using PCA as described previously.

## Results

### Seagrass vulnerability: Mesograzer feeding assays

**Fig 1:**
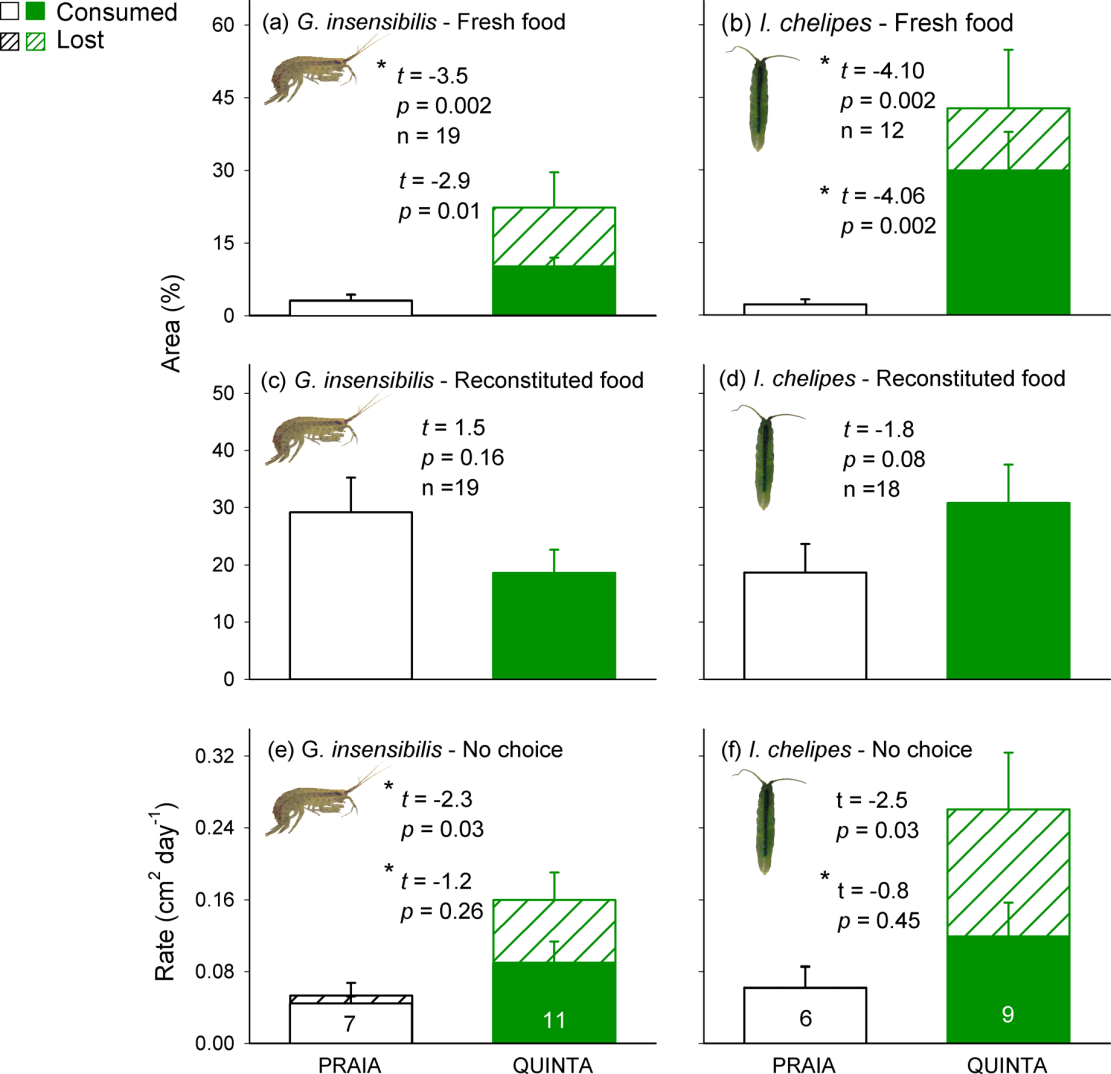
Results of feeding assays examining the vulnerability of *Zostera noltei* to different mesograzers across meadows. Mean (± SE) seagrass consumed (empty bars and statistics below) and lost (= consumed + detached but not consumed; hatched bars and statistics above) by amphipods and isopods in preference assays (fresh material: a, b; reconstituted food: c, d) and no-choice assays (e, f) in which PRAIA and QUINTA plants were offered. Statistics from paired (a-d) and an unpaired (e-f) *t* tests are shown. Sample sizes are shown inside bars for no-choice assays. * Data were log-transformed to meet normality.

*Z*. *noltei* vulnerability in terms of feeding preferences was significantly higher in QUINTA than in PRAIA plants. Both, the amphipod *G*. *insensibilis* and the isopod *I*. *chelipes* showed a strong and consistent preference for QUINTA plants (Fig 1A and 1B). This preference was markedly higher when considering area lost to mesograzers (i.e. consumed plus loose-leaf material), with loose-leaf material appearing only in QUINTA but not in PRAIA plants.

When reconstituted food embedded in agar (i.e. without seagrass structure) was offered, the previously recorded preference of amphipods towards QUINTA leaves in the assays with fresh seagrass disappeared, and we found no significant differences between the consumption of PRAIA and QUINTA artificial food (Fig 1C). We observed a tendency (albeit not significant; P = 0.083) for isopods to maintain the preference towards QUINTA leaves when agar-reconstituted food was offered (Fig 1D), showing not significant differences in effect (preference) size compared to fresh leaves (Mann-Whitney U = 71, p = 0.122). This suggests a combined effect of structural and chemical traits in mediating isopod preference, although relative contribution of chemical/structural traits was lower than 50% as indicated by the effect size with agar-reconstituted food (12.1 ± 6.6), which was less than half the average effect size with fresh food (27.6 ± 8.1; that is 27.6/2 = 13.8).

*Z*. *noltei* vulnerability in terms of mesograzer consumption rates in no-choice assays was not significantly different between PRAIA and QUINTA plants for either amphipods or isopods (Fig 1E and 1F), although averaged area consumed per day was 2-fold higher in QUINTA than in PRAIA plants for both mesograzers. When considering the rate of leaf material loss through bites, the impact of both mesograzers on QUINTA plants was significantly higher than on PRAIA plants, with 2-fold higher area lost than area consumed in QUINTA plants and almost no increase detected in PRAIA plants for both mesograzers.

### Seagrass leaf traits

**Fig 2:**
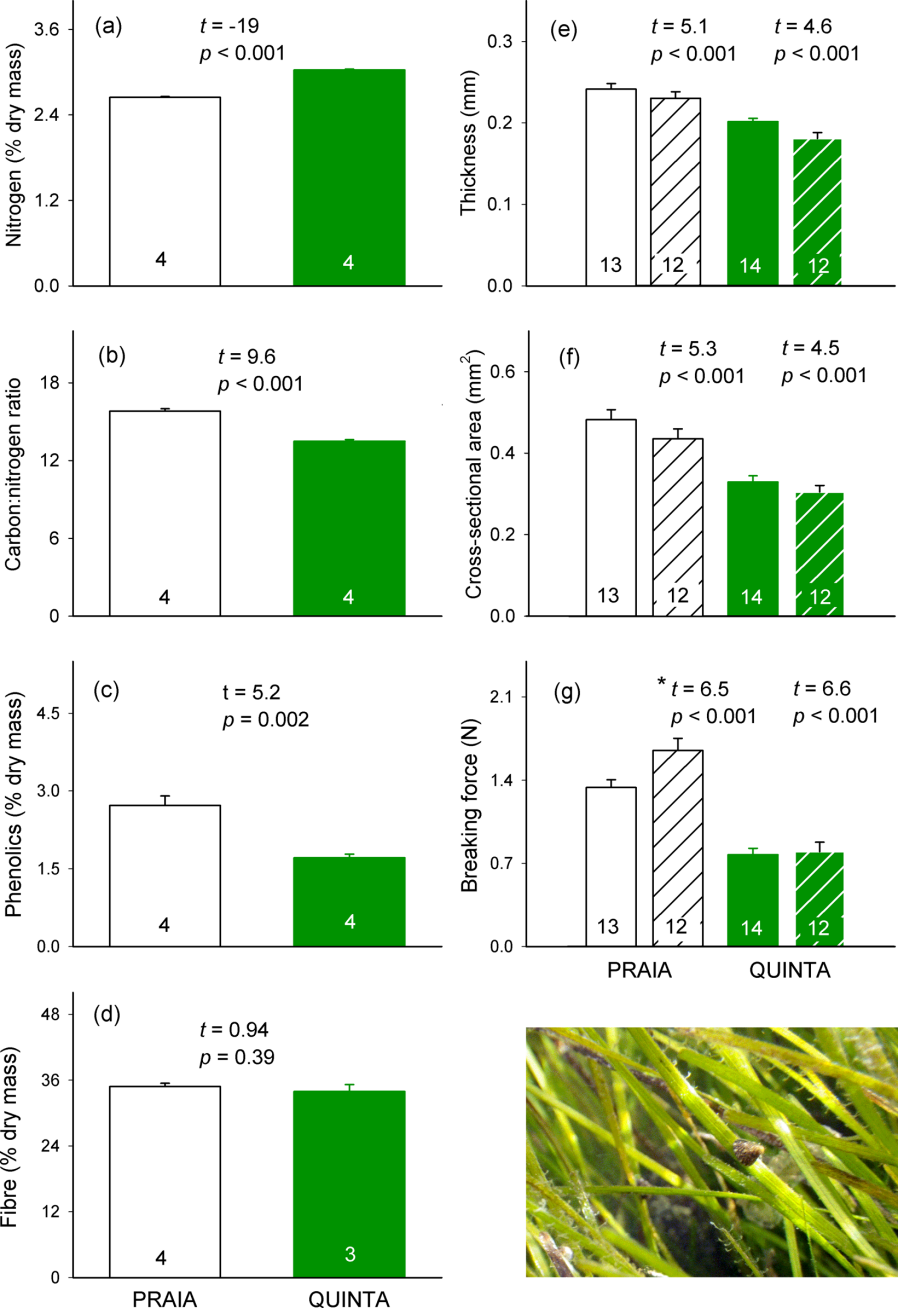
Leaf traits of *Zostera noltei* plants across meadows. Mean (± SE) chemical (a-d) and structural (e-g) traits of *Z*. *noltei* plants from PRAIA and QUINTA meadows. Leaf thickness (e), cross-sectional area (f), and breaking force (g) are shown for fresh (empty bars and statistics on the left) and thawed leaves (hatched bars and statistics on the right). For each leaf trait, statistics from an unpaired *t* test and sample sizes (inside bars) are shown. * Data were log-transformed to meet normality.

All traits of *Z*. *noltei* leaves significantly differed between PRAIA and QUINTA plants, except for fibre content (Fig 2). QUINTA plants exhibited higher leaf nutritional and chemical quality (higher nitrogen content, and lower C:N ratio and phenolic content) and lower structural defences (lowers thickness, cross-sectional area, and breaking force) than PRAIA plants. Significant differences in structural traits between PRAIA and QUINTA plants were equally detected using fresh or thawed leaves (Fig 2E–2G).

**Fig 3:**
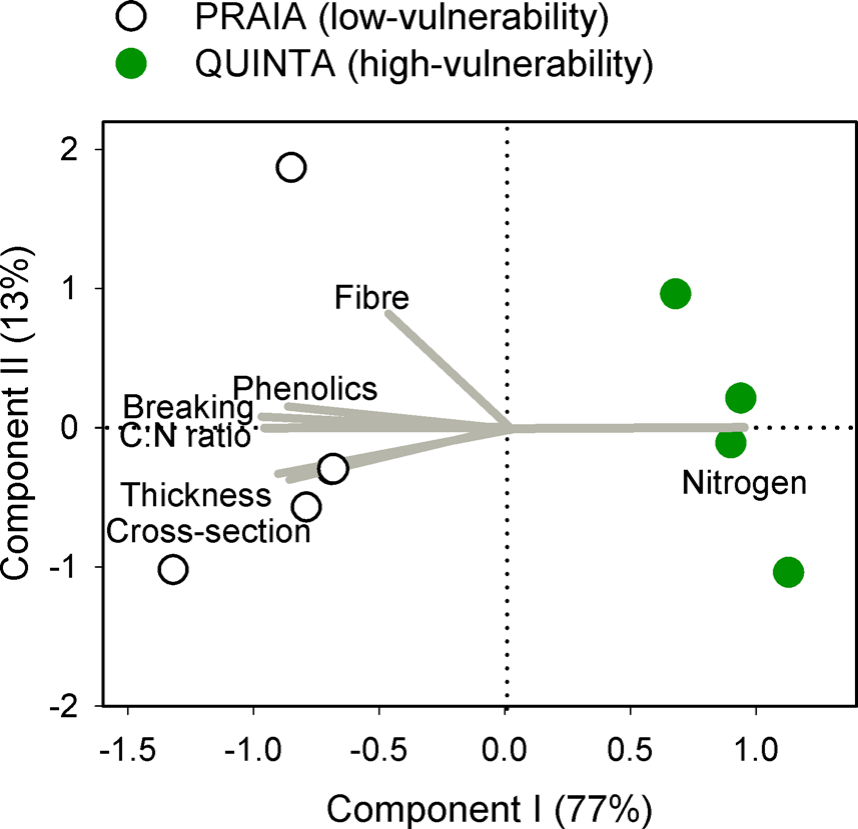
Relationships between leaf traits of *Zostera noltei*. Principal components analyses of *Z*. *noltei* traits in plants with contrasting vulnerability to mesograzers. Trait loadings (grey lines) reflect the correlation to the components and the angles between lines are proportional to their co-variances.

Differences in chemical, nutritional, and structural traits clearly separated PRAIA and QUINTA plants along component I of the PCA, which explained 77% of the variance (Fig 3). All structural defences were positively correlated with each other, while leaf breaking force and thickness also positively correlated with C:N ratio and negatively correlated with nitrogen content (see also correlations in S1 Table and PCA scores in S2 Table). In the case of the cross-sectional area, this correlation was only significant with C:N ratio. A positive correlation with C:N ratio and negative correlation with nitrogen content was also found for phenolics, which also positively correlated with breaking force. Fibre content, which did not correlate to any other trait, was the only variable that significantly correlated with component II of the PCA, which explained only 13% of the total variance. Carbon content did not correlate to any other trait (S1 Table) and was excluded from the PCA analyses.

### Nutrient-enrichment effects on seagrass traits

**Fig 4:**
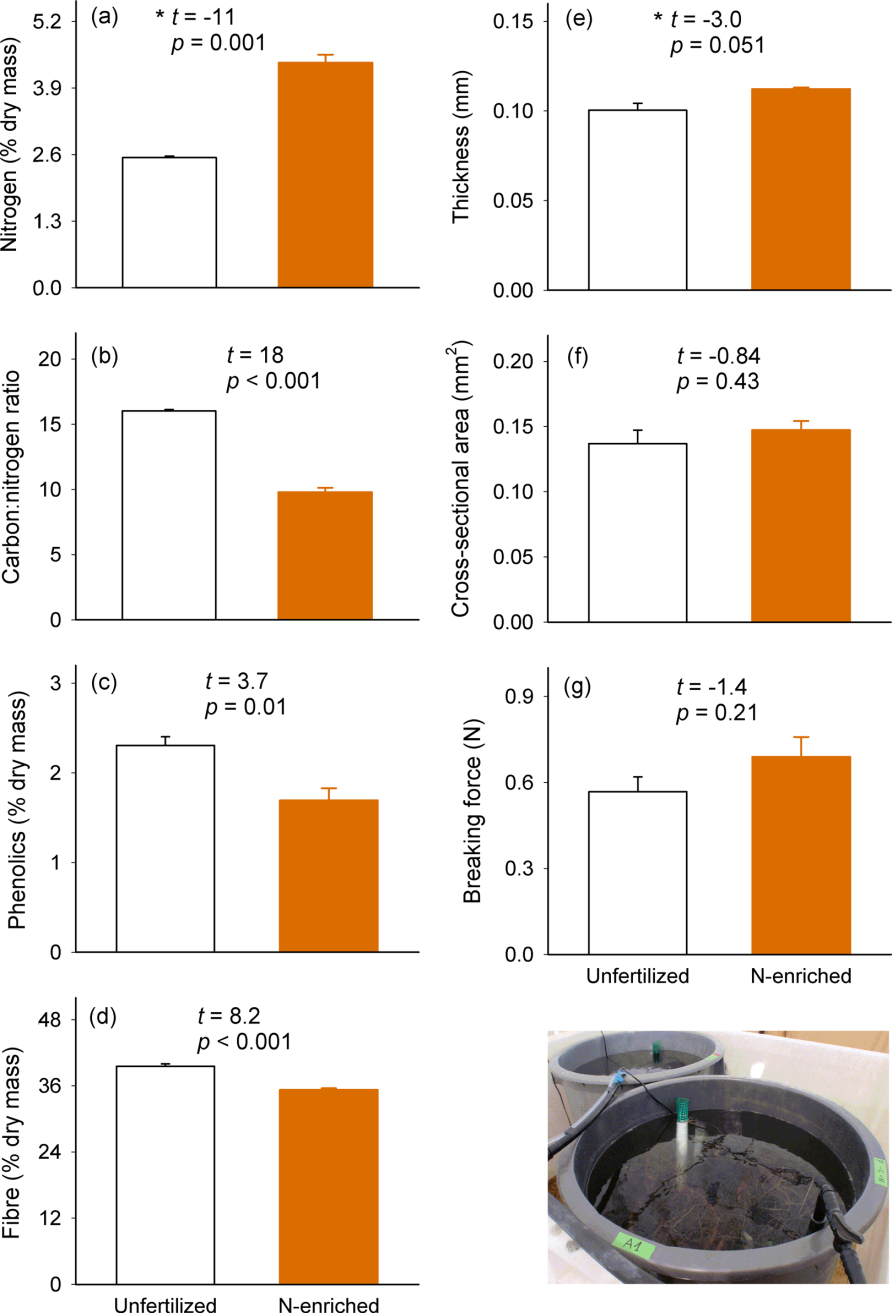
Leaf traits of *Zostera noltei* plants exposed to unfertilized and nutrient-enriched treatments. Mean (± SE) chemical (a-d) and structural (e-g) traits (n = 4). For each leaf trait, statistics from an unpaired *t* test are shown. * Welch´s *t* tests were used when data had unequal variances.

Experimental nutrient enrichment enhanced leaf nutritional and chemical traits by increasing nitrogen content and decreasing C:N ratio, fibre and phenolic content (Fig 4A–4D), while it had no significant effects on structural traits such as leaf thickness, cross section and breaking force (Fig 4E–4G).

**Fig 5:**
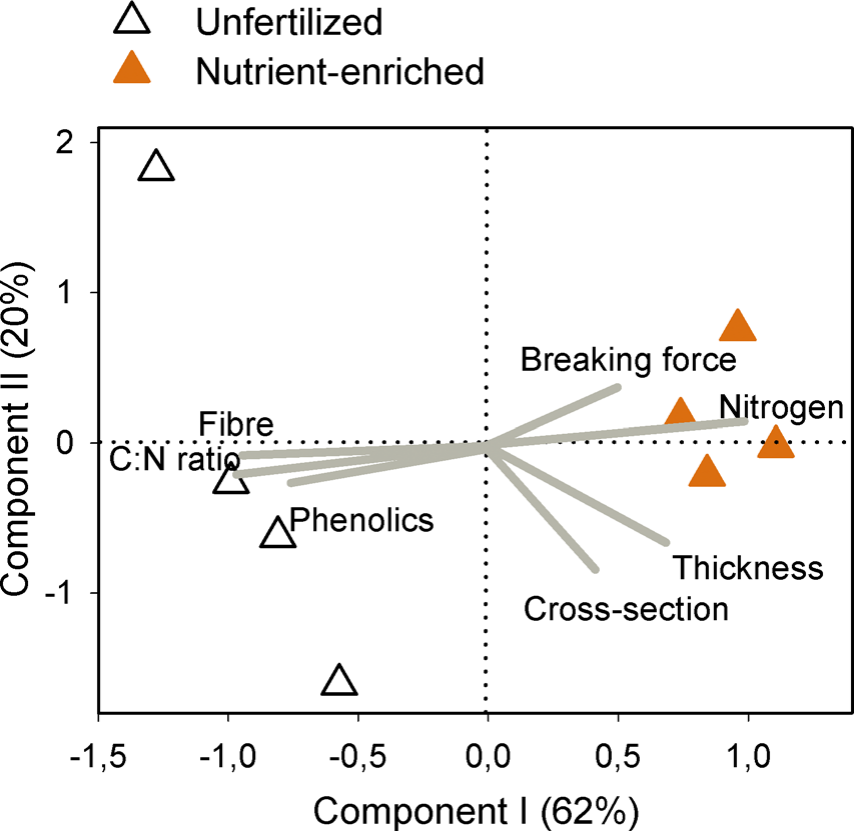
Relationships between leaf traits of *Zostera noltei*. Principal components analyses of *Z*. *noltei* traits in plants exposed to unfertilized and nutrient-enriched treatments. Trait loadings (grey lines) reflect the correlation to the components and the angles between lines are proportional to their co-variances.

Fibre and phenolic contents positively correlated with each other and with C:N ratio, while they negatively correlated with nitrogen content (Fig 5, S3 Table, and S4 Table). Leaf thickness positively correlated with cross-sectional area and nitrogen content, and negatively correlated with fibre content and C:N ratio. Differences in these traits clearly separated unfertilized and nutrient-enriched plants along component I of the PCA, which explained 62% of the variance (Fig 5). Cross-sectional area and, to a lesser extent, leaf thickness highly correlated with component II, which explained 20% of the variance. Unfertilized plants showed a higher dispersal along the along component II than enriched plants. Breaking force did not correlate to any trait. Most correlations of carbon content with other traits matched C:N ratio correlations (S3 Table); thus it was excluded from the PCA analyses in order to avoid redundancy.

## Discussion

It is widely accepted that plant nutritional quality and secondary metabolites play a dominant role in the ecology and evolution of plant defence and that they are strongly linked to vulnerability to herbivores [70, 71]. We found, however, that structural traits have a stronger contribution than chemical traits in mediating *Z*. *noltei* vulnerability to mesograzers. *Z*. *noltei* plants with more tender and thinner leaves were preferentially selected over structurally resistant (i.e. tougher and thicker) plants by both isopods (*I*. *chelipes*) and amphipods (*G*. *insensibilis*). Amphipod feeding preference was strongly influenced by *Z*. *noltei* structural traits, as indicated by the disappearance of the preference towards high-vulnerability (QUINTA) plants when plant structure was removed in the agar-reconstituted food in comparison to the outcomes observed in assays with fresh seagrass. We observed a nearly significant tendency for isopods (p = 0.08) to maintain their preference towards high-vulnerability plants when offered tissues with no plant structure, but the preference size was reduced to less than half compared to fresh seagrass. This indicates a stronger contribution of structural traits (> 50%) but acting in combination with chemical/nutritional traits (< 50% contribution) in mediating isopod preference towards high-vulnerability plants, which had tender and thinner leaves as well as higher nitrogen and lower C:N, fibre, and phenolics. Our results strengthen previous findings of terrestrial studies that suggest that structural rather than chemical traits of plants are the best predictors of plant susceptibility to herbivores [6, 72]. Albeit not statistically significant, the consumption rates by amphipods and isopods of high-vulnerability (QUINTA) plants tended to be higher than of low-vulnerability (PRAIA) plants when offered one type of seagrass tissue. Furthermore, the lower breaking force of the preferred plants likely contributed to the observed increase in mechanical breakdown by mesograzers of leaf material that was not directly consumed, resulting in a significantly higher rate of leaf material loss (i.e. consumed plus loose-leaf material). This breakage and loss of seagrass leaves facilitated by mesograzer injuries may severely amplify the deleterious impact of mesograzer on seagrass production, canopy height, or shoot density as reported by previous studies on *Z*. *noltei* [49] and *Z*. *marina* [51, 73, 74].

Different types of traits involved in plant resistance against herbivory may differently influence the grazing impact and feeding behaviour of co-occurring herbivores (e.g. [57, 75]). Studies that investigate the relative importance of such traits in seagrassses are mostly limited to macrograzers and show important interspecific differences. For instance, structural defences have been reported as primary determinant of sea urchin feeding choices between seagrass tissues [11, 61], while chemical traits mediated intra-specific seagrass preferences of herbivorous fishes [45, 76]. Similarly, structural traits have been reported to determine the discrimination between subtropical seagrass species by omnivorous fishes, while strict herbivorous fishes and urchins seem highly influenced by nutritional traits [77]. Interestingly, we found that different mesograzer species exhibited similar (but not identical) feeding choices and consumption rates, as well as inflicted similar leaf damage in terms of loss of biomass that was not consumed. Previous studies on seaweeds have reported compensatory feeding of mesograzers as a useful strategy to circumvent the effects of food with low nutritional quality for relatively sedentary tube-building species of amphipods [78], while diet mixing seems a more efficient strategy for more mobile non-tube-building amphipods [79]. In agreement with these previous studies, we did not detect any compensatory feeding on *Z*. *noltei* by any of the studied motile mesograzers, as reflected by the similar consumption rates of plants with low and high nitrogen content. Only a non-significant tendency towards higher consumption of nutrient-rich plants was even observed for the amphipod *G*. *insensibilis*. Nonetheless, we are aware that the role of nutritional, chemical and structural traits in determining mesograzer feeding behaviour may differ between seagrass species, as has been pointed out by previous studies [55].

Our study did not detect any overall trade-off between chemical and structural traits in *Z*. *noltei* plants, which contrasts with predictions based on the growth-differentiation balance hypothesis. Our data show that the breaking force of *Z*. *noltei* leaves positively correlates to phenolic content, thus supporting the link between phenolics and structural defences also found by Read et al. [27] in terrestrial systems. According to the defence syndrome hypothesis against herbivory [30], the syndrome of low vulnerability plants corresponds to high defences (structural and phenolics) and low nutritional quality. However, in our study, the role of *Z*. *noltei* phenolics as feeding deterrents was not evidenced for mesograzers. This ambiguous deterrence efficacy of total phenolics is in agreement with previous studies in seagrasses reporting either a preference for seagrass tissues with higher levels of total phenolics by herbivorous urchins [11] and fishes [61] or no effect of increased levels of total phenolics in response to simulated or direct grazing in consumption by isopods or urchins [57, 80]. Furthermore, co-variation of high nitrogen content and low phenolics often found in seagrasses, may confound their effects in mediating herbivore preferences [76]. The relationship between structural defences and phenolics that we observed can be understood at the light of other defensive roles of phenolics. In fact, phenolic compounds are wide-spectrum chemical defences that may act as feeding deterrents, antimicrobials, antioxidants, and UV screens [16, 81, 82], but they can also be incorporated into the lignin of cell walls acting as precursors of structural defences [83, 84]. Our data support a positive association rather than a trade-off between multiple chemical and structural traits across meadows, which has also been found in terrestrial systems [72, 85]. Previous studies suggest that most traits involved in defence have more than one function, which constraints their view as simple alternatives subjected to trade-offs [86, 87]. Our findings are thus in agreement with the increasingly recognized view of plant defences as a suite of co-varying traits, which are physiologically compatible and not mutually exclusive. According to this view, they may act as wide spectrum defences and be the consequence of particular habitat selection pressures and complex underlying factors such as nutrient availability or genetic variability [72, 85, 88]. We also found that leaf breaking force and thickness correlated positively with C:N and negatively with nitrogen content. Our results concur with the correlations between leaf toughness and nutritional traits found by previous studies comprising multiple species of terrestrial plants [89] or seagrasses [65]. On the other hand, leaf fibres are expected to increase the energy required to produce leaf breakage [24], but we found no correlation between fibre content and breaking force. This result could be interpreted in the context of the importance of structural organization and synergy of cell wall components, rather than just contents, in promoting leaf toughness as previously pointed out by Lucas et al. [19]. We also observed that phenolics correlated positively with C:N ratio and negatively with nitrogen content, being this relationship maintained under experimental nutrient enrichment. The correlation that we observed concurs with results of previous studies with few exceptions (see reviews by [28, 90]; and also [76]), and suggests that nutrient deficiency could drive the accumulation of phenolic compounds.

Seagrass exposure to eutrophic nutrient levels markedly increased plant nitrogen content and availability per carbon unit, which reinforces the widely recognized responses of seagrasses following nutrient enrichment (reviewed in [91]). Nutrient enrichment also reduced the accumulation of fibre and total phenolics, with the increase in phenolics being in agreement with previous studies on other seagrass species (e.g. [36, 76]; but see no change in [57]). This result reinforces the aforementioned accumulation of carbon-based compounds under nutrient limitation as predicted by the resource availability hypothesis [92]. Under nutrient enrichment, however, we found no response of leaf breaking force and fine-scale morphology (although Fig 4E suggests a tendency albeit not statistically significant of increased leaf thickness with nutrient enrichment), and their link with nutrient contents was absent except for leaf thickness that positively correlated to nitrogen content and negatively to C:N ratio. Our results indicate that eutrophication is not expected to alter the structural resistance of *Z*. *noltei*, at least in the short-term. These findings contrast with recent studies that show a reduction in leaf breaking force under nutrient enrichment in both, the seagrass *Z*. *noltei* [93] and freshwater plants [94]. They concur, however, with the contrasting response of two fast-growing seagrass species found by La Nafie et al. [95], who detected that under nutrient enrichment *Halophila ovalis* was weaker (but wider and thicker) while *Halodule uninervis* showed no force or thick-morphology response. In our study, chemical and nutritional but not structural traits responded to a short-term nutrient enrichment, thus indicating a higher plasticity of the former compared to the later. Among structural traits, only the obvious positive correlation between cross-sectional area and leaf thickness (used to calculate the former) was observed. Furthermore, no evidence of correlation between chemical and structural traits was detected under nutrient enrichment. Overall, these findings suggest that factors other than a short-term nutrient enrichment were behind the lower structural resistance observed in high- (QUINTA) than in low-vulnerability (PRAIA) plants.

In our study, we did not discriminate between environmental and genetic effects on plant traits, thus the phenotypic correlations between traits tested here are the sum of both genetic and environmental components [96, 97]. For instance, the high genetic diversity that *Z*. *noltei* displays between meadows in the Ria Formosa lagoon (Buga Berković, unpublished data) may point out to genetic variation as a potential explanation of the differences in structural traits that we observed between low- and high-vulnerability plants. Reports of variability in vulnerability to herbivory between different genotypes driven by profound differences on plant traits are available in both, terrestrial plants (e.g. [98]) and seagrasses [36]. Other alternative or complementary explanation can be linked to long-term effects on structural traits of a multi-generational exposure to elevated nutrient levels rather than short-term exposure during the plant life-span. Furthermore, although the two sites in our study were similar in several important environmental variables other than nutrient levels, other abiotic factors that we did not specifically measure (e.g. sediment oxic conditions) may differ between sites and may have also contributed to the observed differences in structural traits between low- and high-vulnerability plants.

## Conclusions

We conclude that intraspecific variation should not be ignored when classifying a single seagrass species with respect to herbivory vulnerability. Our findings reveal that seagrass structural traits such as leaf breaking force and thickness confer mechanical resistance, and hence, protection not only against direct damage and consumption from mesograzers, but also against indirect losses of leaf biomass that is not consumed via breakdown facilitation by mesograzer bites. This protection against breakdown is in agreement with previous studies that reported structural traits to confer mechanical resistance against damage from abiotic factors such as hydrodynamic forces [65, 93, 99]. Seagrass vulnerability (i.e. the loss of structural resistance) was not related, at least in the short-term, to high environmental nutrients as we hypothesized, but was probably linked to seagrass plasticity to other environmental factors that we did not consider and / or to genetic variation between low- and high-vulnerability plants. Importantly, our results did not reveal a trade-off between chemical and structural traits as mutually exclusive defences in *Z*. *noltei* plants. Decreased structural resistance could be an important force contributing to the decline of seagrass meadows and associated species [100] as has been pointed out by previous studies [93].

## Supporting Information

S1 Table Pearson correlation statistics between *Zostera noltei* traits in low- (PRAIA) and high-vulnerability (QUINTA) plants (r above and p-level below; n = 8). *Spearman correlations are shown for traits that had a non-normal distribution even after transformation.
(DOC)

S2 Table Variable loadings in the PCA examining relationships between *Zostera noltei* traits in low- (PRAIA) and high-vulnerability (QUINTA) plants (scaling 2, correlation biplot). (DOC)

S3 Table Pearson correlation statistics between *Zostera noltei* traits in low-vulnerability plants exposed to nutrient enrichment (r above and p-level below; n = 8). *Spearman correlations are shown for traits that had a non-normal distribution even after transformation.
(DOC)

S4 Table Variable loadings in the PCA examining relationships between leaf traits of *Zostera noltei* plants exposed to nutrient enrichment (scaling 2, correlation biplot). (DOC)
