# Supplementary material for: The Role of Seagrass Traits in Mediating Zostera noltei Vulnerability to Mesograzers
Source: PLoS One. 2016 Jun 3;11(6):e0156848. doi: 10.1371/journal.pone.0156848 (PMC4892680; doi:10.1371/journal.pone.0156848)
Supplement: S1 Table — *Spearman correlations are shown for traits that had a non-normal distribution even after transformation. (DOC) [file pone.0156848.s001.doc]

**S1 Table. Pearson correlation statistics between *Zostera noltei* traits in low- (PRAIA) and high-vulnerability (QUINTA) plants** (r above and p-level below; n=8). *Spearman correlations are shown for traits that had a non-normal distribution even after trying log- and sqrt-transformation.

|  | Thickness | Cross-section | Fibre | Phenolics | *Nitrogen | C:N ratio | Carbon |
| --- | --- | --- | --- | --- | --- | --- | --- |
| Breaking | 0.83 | 0.87 | 0.54 | 0.83 | -0.69 | 0.94 | 0.58 |
|  | 0.01 | 0.005 | 0.17 | 0.01 | 0.047 | 0.001 | 0.13 |
|  | Thickness | 0.98 | 0.23 | 0.67 | -0.83 | 0.83 | 0.47 |
|  |  | < 0.0001 | 0.58 | 0.07 | 0.01 | 0.01 | 0.24 |
|  |  | Cross-section | 0.24 | 0.66 | -0.62 | 0.84 | 0.52 |
|  |  |  | 0.57 | 0.08 | 0.09 | 0.01 | 0.19 |
|  |  |  | Fibre | 0.42 | -0.50 | 0.44 | 0.33 |
|  |  |  |  | 0.31 | 0.18 | 0.28 | 0.43 |
|  |  |  |  | Phenolics | -0.83 | 0.80 | 0.20 |
|  |  |  |  |  | 0.01 | 0.02 | 0.63 |
|  |  |  |  |  | *Nitrogen | -0.96 | -0.38 |
|  |  |  |  |  |  | 0.0001 | 0.32 |
|  |  |  |  |  |  | C:N ratio | 0.67 |
|  |  |  |  |  |  |  | 0.07 |
