# Supplementary material for: The Role of Seagrass Traits in Mediating Zostera noltei Vulnerability to Mesograzers
Source: PLoS One. 2016 Jun 3;11(6):e0156848. doi: 10.1371/journal.pone.0156848 (PMC4892680; doi:10.1371/journal.pone.0156848)
Supplement: S2 Table — (DOC) [file pone.0156848.s002.doc]

**S2 Table. Variable loadings in the PCA examining relationships between *Zostera noltei* traits in low- (PRAIA) and high-vulnerability (QUINTA) plants (scaling 2, correlation biplot).**

|  | Component I | Component II |
| --- | --- | --- |
| Phenolics (% dry weight) | -0.87 | 0.10 |
| Nitrogen (% dry weight) | 0.97 | 0.00 |
| C:N ratio | -0.96 | 0.01 |
| Fibre (% dry weight) | -0.48 | 0.83 |
| Breaking force (N) | -0.97 | 0.09 |
| Cross-sectional area (mm^2^) | -0.90 | -0.33 |
| Thickness (mm) | -0.88 | -0.34 |
