# Supplementary material for: The Role of Seagrass Traits in Mediating Zostera noltei Vulnerability to Mesograzers
Source: PLoS One. 2016 Jun 3;11(6):e0156848. doi: 10.1371/journal.pone.0156848 (PMC4892680; doi:10.1371/journal.pone.0156848)
Supplement: S3 Table — *Spearman correlations are shown for traits that had a non-normal distribution even after transformation. (DOC) [file pone.0156848.s003.doc]

**S3 Table. Pearson correlation statistics between *Zostera noltei* traits in low-vulnerability plants exposed to nutrient enrichment** (r above and p-level below; n=8). *Spearman correlations are shown for traits that had a non-normal distribution even after trying log- and sqrt-transformation.

|  | Thickness | Cross-section | Fibre | Phenolics | *Nitrogen | *C:N ratio | Carbon |
| --- | --- | --- | --- | --- | --- | --- | --- |
| Breaking | 0.32 | 0.09 | -0.35 | -0.10 | 0.41 | -0.50 | 0.51 |
|  | 0.44 | 0.83 | 0.40 | 0.82 | 0.29 | 0.18 | 0.20 |
|  | Thickness | 0.72 | -0.78 | -0.58 | 0.86 | -0.83 | 0.71 |
|  |  | 0.046 | 0.02 | 0.14 | 0.002 | 0.005 | 0.048 |
|  |  | Cross-section | -0.41 | -0.04 | 0.38 | -0.33 | 0.58 |
|  |  |  | 0.32 | 0.93 | 0.32 | 0.39 | 0.14 |
|  |  |  | Fibre | 0.77 | -0.86 | 0.79 | -0.76 |
|  |  |  |  | 0.03 | 0.002 | 0.01 | 0.03 |
|  |  |  |  | Phenolics | -0.71 | 0.76 | -0.58 |
|  |  |  |  |  | 0.04 | 0.02 | 0.13 |
|  |  |  |  |  | *Nitrogen | -0.98 | 0.91 |
|  |  |  |  |  |  | < 0.0001 | < 0.0001 |
|  |  |  |  |  |  | *C:N ratio | -0.86 |
|  |  |  |  |  |  |  | 0.002 |
