# Supplementary material for: The Role of Seagrass Traits in Mediating Zostera noltei Vulnerability to Mesograzers
Source: PLoS One. 2016 Jun 3;11(6):e0156848. doi: 10.1371/journal.pone.0156848 (PMC4892680; doi:10.1371/journal.pone.0156848)
Supplement: S4 Table — (DOC) [file pone.0156848.s004.doc]

**S4 Table. Variable loadings in the PCA examining relationships between leaf traits of *Zostera noltei* plants exposed to nutrient enrichment** (scaling 2, correlation biplot).

|  | Component I | Component II |
| --- | --- | --- |
| Phenolics (% dry weight) | -0.77 | -0.25 |
| Nitrogen (% dry weight) | 0.98 | 0.17 |
| C:N ratio | -0.98 | -0.19 |
| Fibre (% dry weight) | -0.96 | -0.04 |
| Breaking force (N) | 0.50 | 0.41 |
| Cross-sectional area (mm^2^) | 0.42 | -0.84 |
| Thickness (mm) | 0.68 | -0.65 |
